# The New Anodyne

**Published:** 1871-02

**Authors:** 


### THE NEW ANODYNE.

The medical world is delighted with the discovery of a new medicine, and the newspapers abound with advertisements, setting forth the peculiar advantages of each preparation, every man claiming that his own is the best. It is called —

### CHLORAL HYDRATE.

The object of taking this medicine is to promote sleep, and thus far, when administered pure and in a proper manner, it has advantages above all others known hitherto, whether in the form of Opium, Morphine, Laudanum, or Paregoric.

This Chloral Hydrate is in white crystals; almost every liquid dissolves it; hence it is largely advertised in the shape of syrups, anodynes, and various fluid mixtures. When it is dissolved in any fluid, it begins to lose its peculiar power within an hour; hence it should be mixed and used on the spot. Therefore, if employed at all, each person should purchase it in its white crystal form direct from the apothecary, and mix it himself; then only can he get all its advantages, and know what and how much he is taking; all preparations of Chloral Hydrate other than in white crystal form are gross impositions on the public.

There can be no doubt of its advantageous use under the physician's care for a few times; but any one who takes it on his own responsibility, especially if used night after night for promoting sleep, acts most unwisely, for it has not been in use long enough to allow proper observations to be made as to its safety. There are several medicines which can be taken a few times without any apparent ill result; but if their employment is continued, the most ineradicable and deathly consequences follow. A man may drink water brought into the house by a faulty lead pipe, and notice no inconvenience; he may repeat that drinking for days and weeks, and be apparently as well as ever; but continuing to use it for months, the most incurable poisoning follows, and in multitudes of cases fatal, after months and years of torment.

All anodynes act more or less directly on the brain, the most delicate and dangerous part of the whole body to be tampered with, and Chloral may have its far-reaching tendencies there to impair and to destroy. Sleep healthful, delicious, and invigorating, as more fully treated in the book on "Sleep," or

"THE HYGYENE OF THE NIGHT,"

comes only from the exhaustion to a certain point of that store of strength with which we leave our chambers in the morning, which "store of strength" is the result of rest to body and brain. This store of strength comes up, accumulates as naturally as the water accumulates in a well which has been left almost dry from excessive use; the water rises higher and higher, simply from the well being let alone. So bodily strength rises up, as it were, during a state of repose, while asleep at night, and does this more favorably if let alone to its own natural actions. The best and only healthful anodyne in the world, the most unobjectionable sleep-producer, is sturdy, honest out-door labor; for the Bible has said it: "The sleep of a laboring man is sweet."

